# Global research productions pertaining to atrial fibrillation from 2004 to 2018: A bibliometric analysis

**DOI:** 10.1097/MD.0000000000018971

**Published:** 2020-01-31

**Authors:** Shuqing Shi, Jingjing Shi, Shuai Shi, Qiulei Jia, Guozhen Yuan, Yi Wei, Dandan Wang, Yuanhui Hu

**Affiliations:** a Graduate School, Beijing University of Chinese Medicine; b Department of cardiovascular, Guang’anmen Hospital, China Academy of Chinese Medical Sciences, Beijing, China.

**Keywords:** atrial fibrillation, bibliometric analysis, CiteSpace, research hotspots, VOS viewer

## Abstract

**Background:**

We analyzed the scientific outputs of global atrial fibrillation (AF) researches, developed a model to qualitatively and quantitatively evaluate the AF research productions from 2004 to 2018.

**Methods:**

The data was retrieved from the Web of Science Core Collection (WOSCC) on June 15, 2019. Bibliometrics tools—CiteSpace V (Drexel university, Chaomei Chen) and VOSviewer (Leiden University, van Eck NJ) --were used for bibliometric analyzing published outputs and finding research hotspots.

**Results:**

There were a total of 21,839 research articles on AF, and the annual publication rate increased over time from 2004 to 2018. The *Journal of Cardiovascular Electrophysiology* published the greatest number of articles, and the leading country was the United States. The leading institution was the Mayo Clinic, and the most productive researchers were: LIP GYH, Natale A, Chen SA, DI Biase L, and Kuck KH. The keywords analysis showed that catheter ablation, risk, heart failure, stroke, and management were research hotspots.

**Conclusion:**

Bibliometric analysis of the literature shows that research on AF continues to be a hot spot, and the clinical treatment of AF is an important research frontier. However, further research and collaboration are needed worldwide. Our findings aim to provide valuable information for the editors of journals that publish research on arrhythmia, and to help researchers identify new perspectives for future researches.

## 1 Introduction

Atrial fibrillation (AF) occurs when the atriums do not coordinate with the conduction of impulses, which results in irregular fibrillation of atrial muscle fibers and loss of effective mechanical contractions. According to a recent research, there are 20.9 million male and 12.6 million female patients with AF (excluding asymptomatic patients).^[1]^ There will be 14 to 17 million patients with AF in the European Union by 2030, with an annual increase of 120 to 215 thousand new patients.^[2]^ And there will be about 5.2 million in the United States, which will increase to 12.1 million by 2030.^[3]^ The increasing prevalence of AF has made it a global concern.

The progression of AF is often associated with high mortality and disability risk.^[4]^ The incidence of stroke in patients with AF is much higher than in those without AF ^[5–7]^ and sudden cardiac death associated with AF contributes to the high mortality.^[8,9]^ Due to its widespread prevalence and potentially serious complications, AF puts a huge economic burden on the public health and medical systems in both developed and developing countries. It has been estimated that AF accounted for about 1% of the UK National Health Service's (NHS) budget, and $16 to 26 billion has been spent on AF health care in the United States per year.^[10–12]^

Over the past 15 years, a large number of new discoveries on AF have been made and published by researchers, indicating continuous progress in this field. However, these researches have not been analyzed and mapped systematically, with only a few bibliometric studies focused on cardiovascular disease. ^[13–15]^ Our bibliometrics study provides an overview of these outputs and identifies the influential countries, institutions, authors, publications, and milestone theories. We hope our results could contribute to the development of AF research and give some suggestions to the policy makers, researchers, and funding agencies that work on AF researches and treatments.

## 2 Methods and materials

### 2.1 Data sources and retrieval strategies

For this bibliometric analysis, we selected the Web of Science Core Collection (WOSCC) of Thomson Reuters as our database. The search parameters were: (Atrial Fibrillations OR Auricular Fibrillation OR Persistent Atrial Fibrillation OR Familial Atrial Fibrillations OR Paroxysmal Atrial Fibrillation); time span: 2004 to 2018; category refined: Cardiac Cardiovascular Systems; literature type: Article; index: sci-expanded. No language restrictions. All records (include titles, authors, sources, abstracts) and references in our search results were exported in plain text format. Retrieval work was performed in 1 day (on June 15, 2019) to avoid variations due to daily updates to the database. The data is all secondary data and does not contain any personal information, therefore informed consent was not required.

### 2.2 Methods

Excel 2019(Redmond, WA), CiteSpace V (Version 5.3 R4, Drexel university, Chaomei Chen), ^[16]^ and VOSviewer (Version 1.6.11, Leiden University, van EckNJ) ^[17]^ were used for bibliometric analysis of the data we retrieved. Excel 2019 was used to display the number of published articles and the journal distribution trends in each year. CiteSpace V was used for the co-authorship network of Countries/Institutions/Authors, dual-map analysis and co-occurrence of keywords. To understand the research frontiers, burst detection was applied to investigate the growth rate of citations of keywords using CiteSpace. The co-authorship/co-occurrence maps consists of nodes representing elements such as countries, institutions, authors, and keywords, as well as links between nodes representing co-author relationships and co-occurrence relationships.^[18]^ The color of the links between the nodes represents the year of the first common reference. Centrality is an indicator used to measure the importance of an element. If the centrality is more than 0.1, the element is considered relatively important and represented by a purple ring.^[19]^ The VOSviewer was used for co-citation analysis of References/Journals/Authors to construct maps of relevant knowledge. In the co-citation maps, different nodes representing elements such as cited references, cited journals and cited authors, and the size of nodes reflect the number or frequency of citations.^[17]^ The links between nodes represent co-citation relationships.^[18,20]^ The colors of nodes and lines indicate different clusters or years.^[21]^ The impact factor of each journal was derived from the 2019 journal citation report (JCR) (Clarivate Analytics, Philadelphia, USA).

CiteSpace parameters were used as follows: time slice (2004–2018), year of each slice (1), term source (all selected), node type (1 at a time), selection criteria (50), pruning (Pathfinder), and visualization (cluster viewstatic, display merged network). The fractional counting method in VOSviewer was used and the chosen threshold was set to select publications. Finally, the top 100 cited references/journals/authors were selected.

## 3 Results

### 3.1 General publication outputs

**Figure 1:**
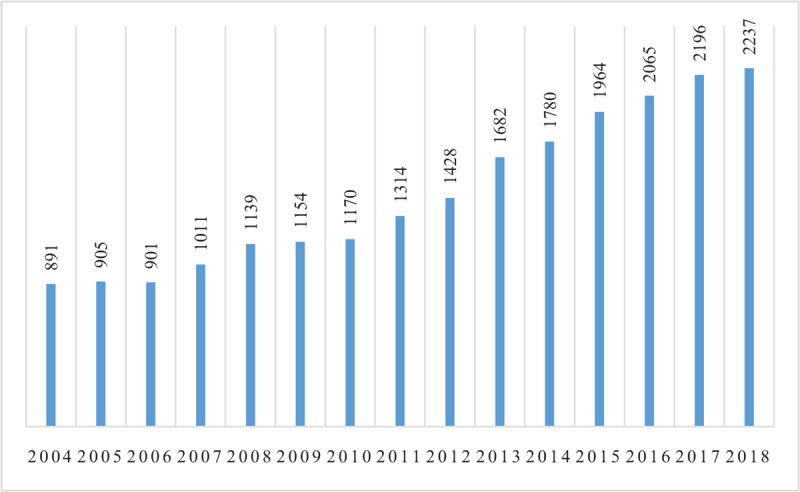
The annual number of publications on AF indexed in WOSCC from 2004 to 2018.

Based on the selection criteria, 21,852 publications of AF in WOSCC from 2004 to 2018 were indexed. After duplicates (12) and retraction (1) removed, there were a total of 21,839 publications left (Fig. 1). From 891 articles in 2004 to 2237 in 2018, we can see that the number of publications per year was constantly increasing (Fig. 1). Between 2004 and 2006, the number of publications increased slightly, with annual publications less than 1000. After 2007, the number of publications grew rapidly, with more than 1000 articles published per year. After 2016, the number of publications exceeded 2000 each year and reached the peaked in 2018.

The total 21,839 articles were published in 10 languages, English was the most widely used language (20,889, or 95.650%). The other articles were published in following languages: 389 (1.781%) in Russian, 135 (0.618%) in German, 123 (0.563%) in Spanish, 96 (0.440%) in Portuguese, 81 (0.371%) in French, 64 (0.293%) in Polish, 37 (0.169%) in Italian, and 22 (0.101%) in Turkish.

The total 21,839 articles were published in 193 journals (Table 1). Table 1 shows the top 15 most popular journals for publishing articles on AF. They published 11,513 articles and accounted for 52.72% of all articles. The *Journal of Cardiovascular Electrophysiology* had the largest number of published articles (1,401, 6.415%), followed by *Euro Pace* (1,357, 6.214%), *Heart Rhythm* (1,195, 5.472%), *International Journal of Cardiology (*1,024, 4.689%), and *American Journal of Cardiology* (991, 4.538%).

**Table 1:**
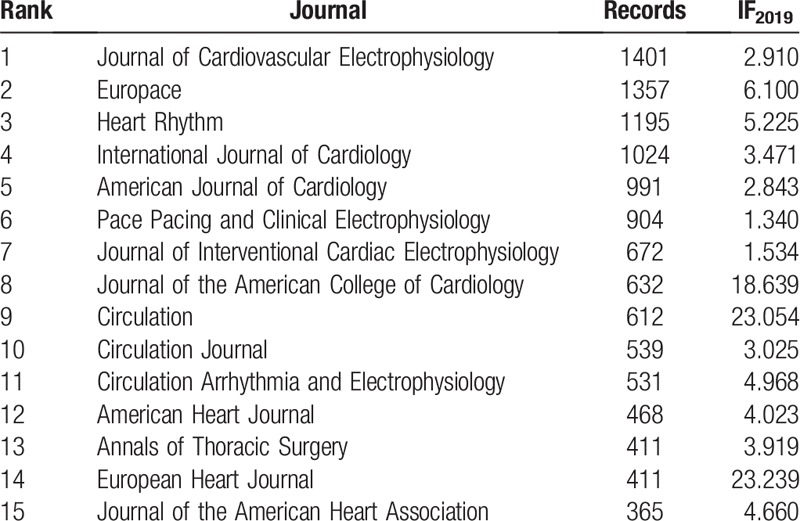
The total numbers and IF_2019_ of top 15 popular journals.

### 3.2 Co-authorship: countries, institutions, and authors

A total of 125 countries and regions made contributions to publications on AF research (Tables 2 and 3, Fig. 2 .A). The USA (5249 papers), Germany (1853 papers), Japan (1524 papers), Italy (1340 papers) and England (1217 papers) were the top 5 productive countries. In terms of centrality, the top 5 countries were Netherlands (0.4), Scotland (0.36), the United States (0.28), Canada (0.2), and Italy (0.16).

**Table 2:**
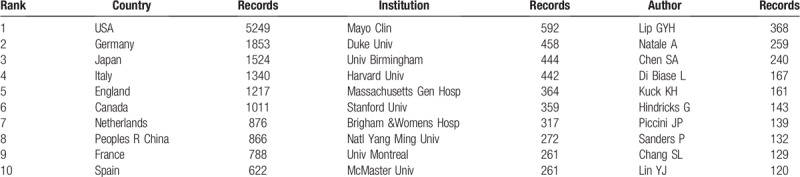
The top 10 active countries, institutions and authors.

**Table 3:**
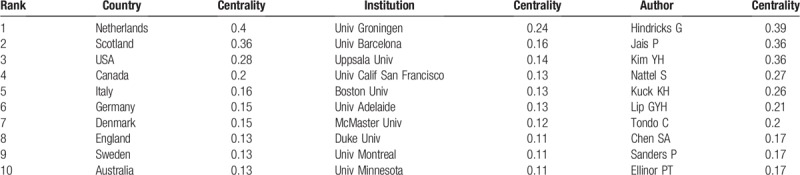
The Top 10 Countries, Institutions and Authors with high Centrality value.

**Figure 2:**
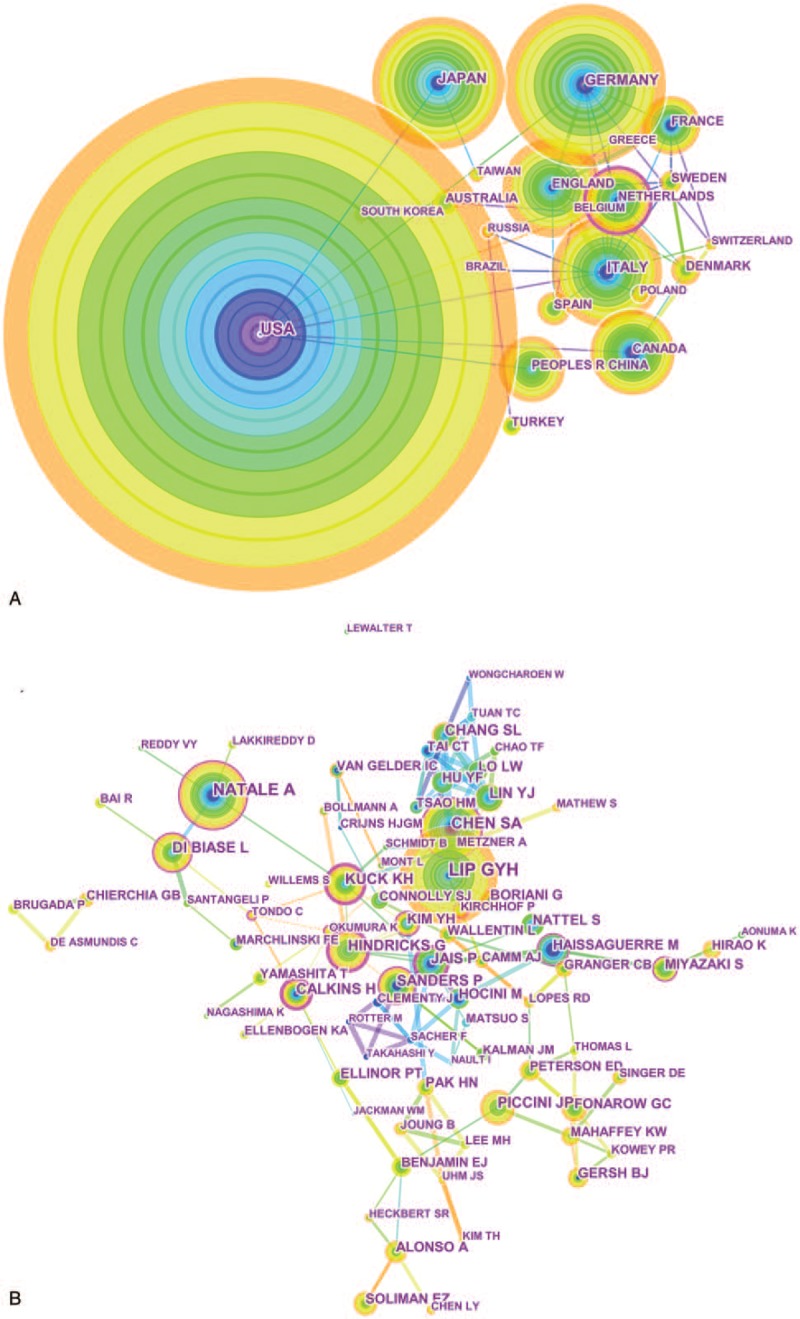
A. Co-authorship between countries with more than 300 publications. B. Co-authorship between institutions with more than 100 publications. C. Co-authorship between among authors with more than 30 publications.

**Figure 2 (Continued):**
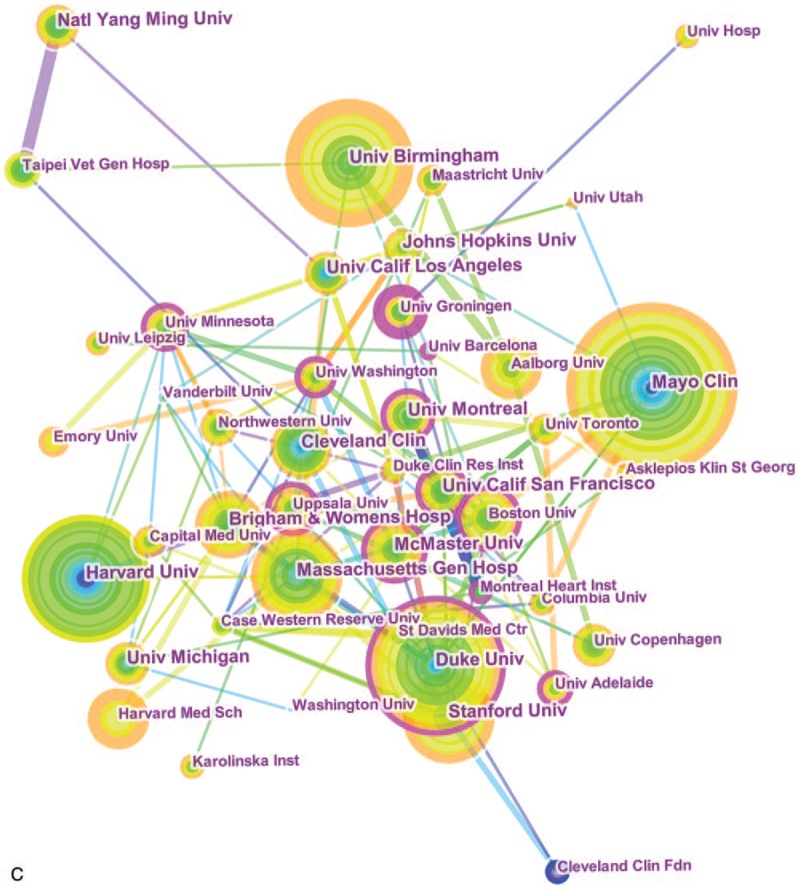
A. Co-authorship between countries with more than 300 publications. B. Co-authorship between institutions with more than 100 publications. C. Co-authorship between among authors with more than 30 publications.

The 21,839 articles were mainly contributed by 13,168 institutions (Fig. 2 .B). As shown in Table 2, The Mayo Clinic (592 papers) was the most productive institution, followed by Duke University (458 papers), University of Birmingham (444 papers), Harvard University (442 papers), and Massachusetts General Hospital (364 papers). The 5 institutions with the highest centrality value were University of Groningen (0.24), University of Barcelona (0.16), Uppsala University (0.14), University of California at San Francisco (0.13), and Boston University (0.13).

The 21,839 articles were written by 71,864 authors (Fig. 2 .C). The top 10 most active authors are shown in Table 2, Gregory YH Lip (368 papers) from the Institute of Applied Health Research University of Birmingham United Kingdom ranked first, followed by Natale A (259 papers), Chen SA (240 papers), DI Biase L (167 papers), and Kuck KH (161papers).

### 3.3 Co-citation: references, journals, and authors

Co-cited references refers to references cited by other 2 papers simultaneously.^[22,23]^ The link indicates that there was a co-citation relationship between the 2 references. Co-cited authors and co-cited journals were derived from the co-cited reference. Furthermore, to measure the influence of co-cited references/journals/authors. The centrality values of references/journals/authors were abstained by means of CiteSpace (Table 5).

**Figure 3:**
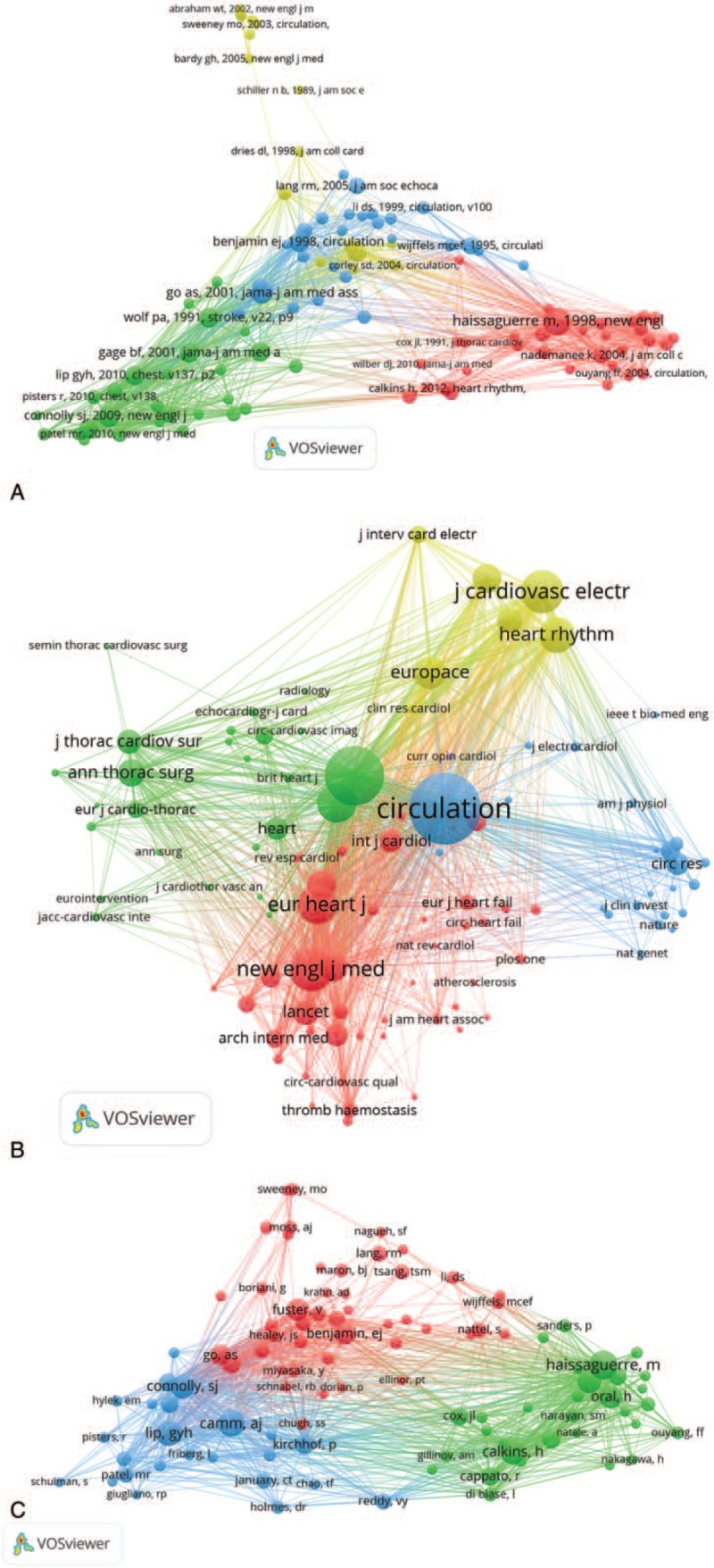
A. Map of Co-Citations for Top 100 Most Cited References. B. Map Co-Citations for Top 100 Most Cited Journals. C. Map Co-Citations for Top 100 Most Cited Authors.

A total of 192,799 references were cited in 21,839 articles according to VOSviewer (Fig. 3.A). The top 10 cited references on AF research were cited more than 500 times each. In these references, 5 were cited more than 600 times, and 3 were cited more than 900 times,

In total, 15,805 journals were cited in 21,839 articles (Fig. 3.B). In the top 10 cited journals (Table 4), *Circulation* (18,658 records) ranked first, followed by *J Am Coll Cardiol* (16,745 records), *Eur Heart J*(11,316 records), *New Engl J Med*(11,309 records), and *Am J Cardiol*(10,871records).

**Table 4:**
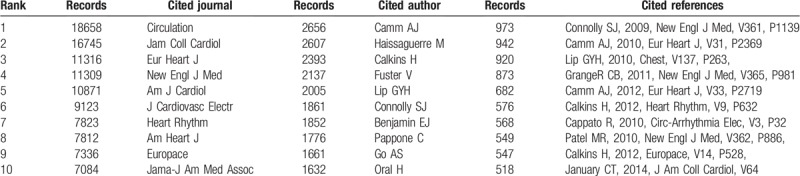
The top 10 journals, authors and references with the highest citations.

**Table 5:**
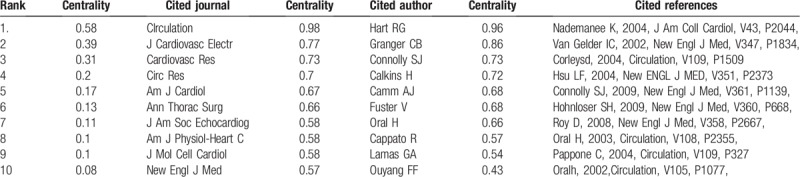
Top 10 cited journals, authors and references with highest centrality value.

92,877 cited authors were cited in 21,839 articles (Fig. 3.C). Table 4 presents the top 10 cited authors on AF research. Among them, CAMM AJ (2656 records) ranked first, followed by HAISSAGUERRE M (2607 records), CALKINS H (2393 records), FUSTER V (2137 records), and LIP GYH (2005 records).

**Figure 4:**
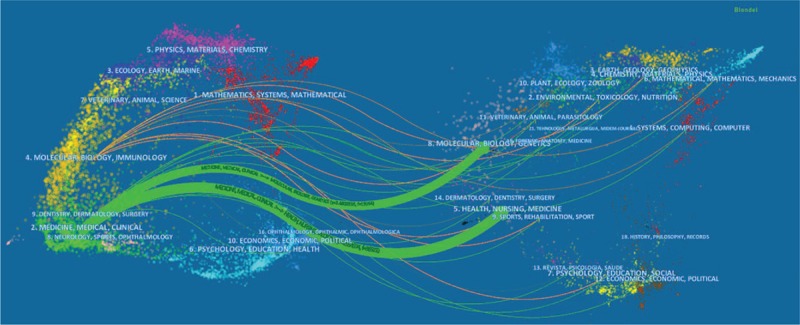
Dual-map overlay of journals related to AF.

Figure 4 was a dual-map overlay of journals. On the left was the citing journals map, and on the right was the cited journals map.^[24]^ Most of the papers were published in the field of “Medicine, Medical and Clinical” shown on the left in Figure 4, and these were mainly influenced by the field of “Molecular, Biology, Genetics, and Health” shown on the right. Journal topics were labeled, with citation paths visualized as colored curves, showing that there were 2 main citation paths in the current map.

### 3.4 Co-occurrence analysis—keywords

**Figure 5:**
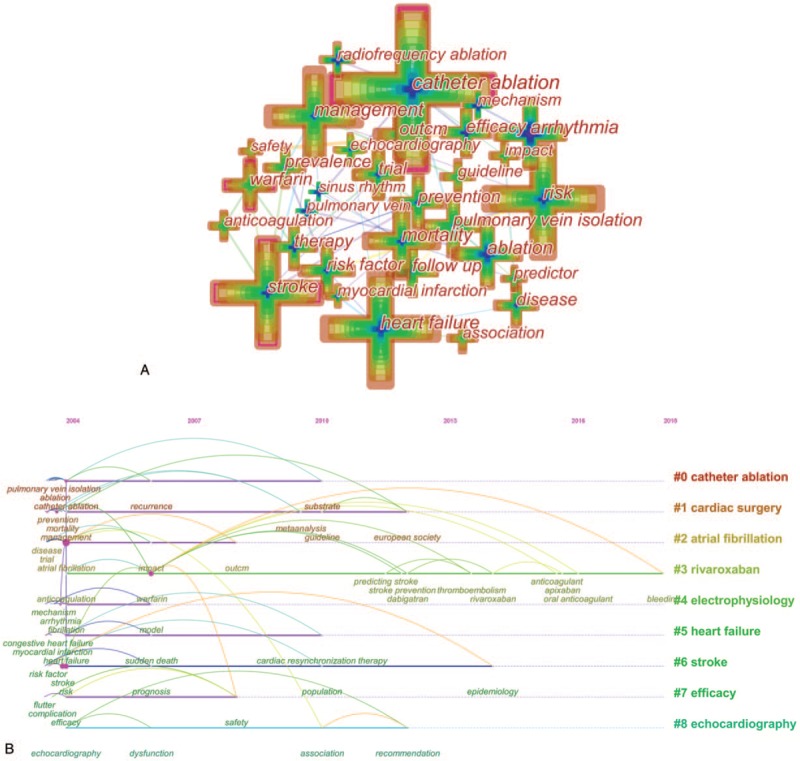
A. Keywords Co-occurrence map of publications on AF. B. The timeline view of Keyword clusters of publications on AF.

According to our study, the top foci of AF research resulted in 87 nodes and 186 links when we generated a keywords co-occurrence map (Fig. 5.A). The top 10 keywords used more than 2000 times were as follows: atrial fibrillation (12,559records), catheter ablation (3808 records), risk (2752 records), heart failure (2744records), fibrillation (2549 records), stroke (2523 records), and management (2261 records) (Table 6).

**Table 6:**
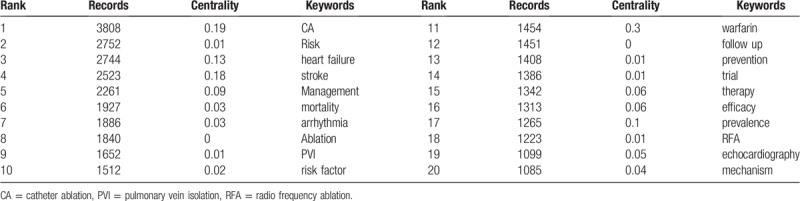
Top 20 keywords in terms of records and centrality in AF research.

Log-likelihood ratio algorithm was used to aggregate 87 keywords to obtain 9 clusters. The silhouette value of clusters 0 to 8 was from 0.679 to 0.895, showing good homogeneity^[18]^ All clusters were labeled by index terms extracted from the keywords. The largest cluster (#0) was labeled as “catheter ablation”, followed by the second largest cluster (#1) labeled as “cardiac surgery”, and the third largest cluster (#2) was labeled as “atrial fibrillation”. These clusters were also shown in a timeline view (Fig. 5.B).

In further analysis, CiteSpace was used to detect the keywords with the strongest citation bursts, in order to find the research frontier of AF (Fig. 6 ). The keywords with the strongest citation bursts after 2016 were listed as follows: oral anticoagulant (84.503), registry (83.3768), ischemic stroke (78.4979), cardiovascular disease (78.1227), apixaban (70.6214), anticoagulant (65.7484), association (47.4639), and meta-analysis (42.0579).

**Figure 6:**
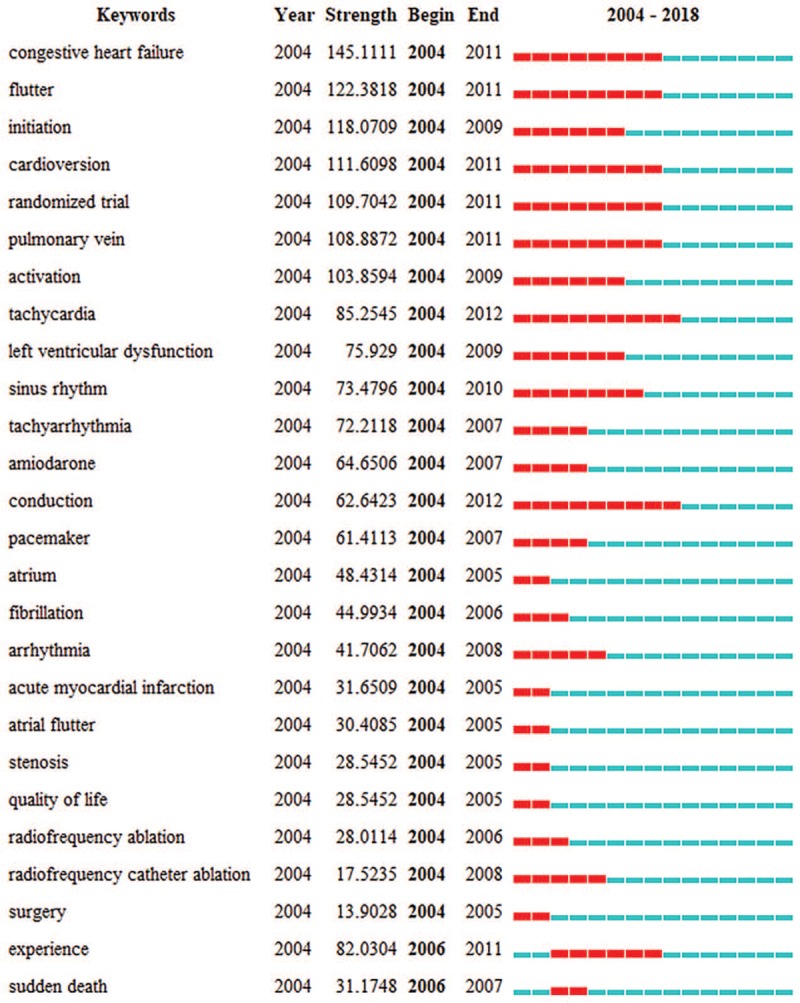
(1). Top 54 Keywords with the strongest citation bursts. (2). Top 54 Keywords with the strongest citation bursts.

**Figure 6 (Continued):**
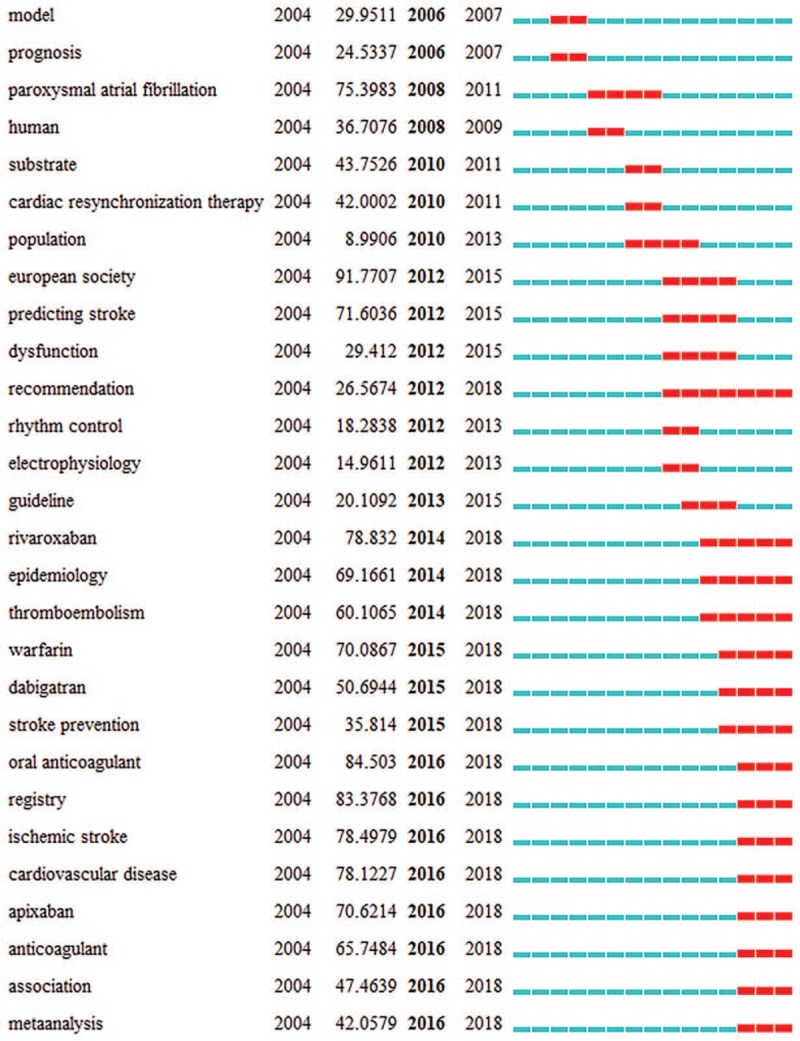
(1). Top 54 Keywords with the strongest citation bursts. (2). Top 54 Keywords with the strongest citation bursts.

## 4 Discussion

### 4.1 General data

Analysis of the top 15 most popular journals shows 20% (3/15) of the journals had an impact factor greater than 10. These journals were *Journal of The American College of Cardiology* (IF_2019_ = 18.639), *Circulation* (IF_2019_ = 23.054), and *European Heart Journal* (IF_2019_ = 23.239). About 13.33% (2/15) had an impact factor between 5 and 10, including the *Euro pace* (IF_2019_ = 6.100) and *Heart Rhythm* (IF_2019_ = 5.225). 40% (6/15) had an impact factor between 3 and 5, 26.67% (4/15) had an impact factor lower than 3. In summary, publications in the AF field were mainly published on middle-high IF journals(IF>3,73.33%,11/15) and it was still a challenge to publish papers about AF in high IF journals (IF>10, 20%,3/15).

We speculate that scholars on AF choose journals where their papers are most likely to be accepted. At the same time, they searched publications in cutting-edge research journals. Articles use new scientific research technologies, and provided significant evidence for clinical practices were most easily accepted.

### 4.2 Co-authorship: countries, institutions, and authors

Co-operations between countries played a vital role in the development of AF researches. The top 10 countries (Table 1) included 6 European countries, 2 North American countries and 2 Asian countries. The combined outputs of these 10 countries were 15,346, accounting for 70.97% of the total outputs. European and North American countries leaded the AF research, especially the United States, which took the core position in global AF research and cooperated closely with various countries Netherlands ranked seventh in terms of numbers of publications, but had the highest value of centrality, indicating high research quality and great influence. China was the only developing country among the top 10 countries in the number of articles, which indicated that China has made great progress on AF in the past 15 years. Among the 125 countries, only 3 in South American (Brazil, Chile and Venezuela). The collective outputs of Africa was less than 100 papers. It is clear that the highest outputs of research on AF come from institutions in developed countries. Low- and middle-income countries lag far behind developed countries in medical care and scientific research and need to strengthen international cooperation.

The top 10 institutions with the most published articles accounted for 13.47% of the total published articles. The global distribution of institutions was consistent with the distribution of countries. Among the top 10 institutions, 6 from the United States, 2 from Canada, 1 from the United Kingdom, and 1 from Singapore. Among these 10 institutions, 3 were hospitals, and 7 were universities. The Mayo Clinic, Duke University, the University of Birmingham, Harvard University, and Massachusetts General Hospital were leading institutions in AF research. Figure 1B shows that there are many links between institutions, those had close cooperative relationships are indicated by relatively thick lines. We conclude that the research results of these institutions can reflect both their level of research, scholarship, academic exchanges, and cooperative projects. It is worth noting that cooperation between institutions was not limited by geography. Moreover, the rankings for the number of posts were not the same as the rankings for centrality, which measured significance. For example, Univ Groningen of the Netherlands ranked 23rd in terms of the number of articles (193 papers), but ranked first in centrality, which indicated that his research publications had both great influence and high quality. We estimate that the leading position of the Netherlands in AF was mainly due to Univ Groningen's research in the epidemiology of AF.^[1]^

There were 14 authors with more than 100 published papers who can be called prolific authors.^[25]^ However, in this group, the centrality of Piccini JP, Lin YJ, Gersh BJ, and Haissaguerre M were all lower than 0.1, indicating that prolific authors should put more emphasis on the quality of articles, not just the quantity. Looking at the authors research direction and level of institutional cooperation, these could be roughly divided into 4 major academic groups:

1. the key members of the academic group 1 were Piccini JP, Gersh BJ, and Sanders, P. This group was mainly engaged in radio frequency ablation (RFA) and linear ablation surgical treatment of AF;
2. the key members of the academic group 2 were Natale A, Di Biase L, and Hindricks G. They mainly engaged in the separation of the pulmonary vein cavity;
3. the main members of the academic group 3 were Lip GYH, Soliman EZ, and Alonso A, who mainly engaged in the epidemiology of AF;
4. the main members of academic group 4 included Chen SA, Chang SL, and Lin YJ, who mainly engaged in research on AF electrophysiology.

### 4.3 Co-citation: references, journals, and authors

Through the analysis of the ranking of citation frequency and centrality, we found 5 “classic texts”. These texts had laid a theoretical and practical foundation for related research of AF, so we can call them landmark studies. The most frequent co-cited reference was published in 2009 by Connolly et al. This was the first large-scale study to compare the effects of 2 doses of dabigatron and warfarin on stroke risk in patients with AF and to identify effective strategies for preventing stroke and systemic embolism with AF.^[26]^ The second most frequently co-cited reference was the 2010 ESC guidelines for the managements of AF. The guidelines proposed a new scoring system--CHA2DS2VASC score, which changed the score from 1 to 2 for age ≥75 years on the basis of CHADS2 score, and added 3 risk factors including vascular disease, people age 65 to 74 years, and gender (female) with a maximum score of 9.^[27]^ The third most frequently co-cited reference was published by Lip et al in 2010. In this paper, the authors proposed a simple and novel model for improving stroke risk stratification in patients with AF, and the real value of this model was demonstrated by the Birmingham 2009 risk trial in AF populations of different race/ethnicity.^[28]^ The paper with the highest centrality value was published in 2004 by Nadenanee K et al. This study discovered and verified that areas with complex fractionated electrograms (CFAEs) represent a defined electrophysiologic substrate and were ideal target sites for ablations to eliminate AF and restore normal sinus rhythm.^[29]^ The paper with the second highest centrality value was published in 2002 by Van Gelder et al. This study illustrated that rate control was not inferior to rhythm control for the prevention of death and morbidity from cardiovascular causes. Therefore, it may be an appropriate therapy for patients with a recurrence of persistent AF after electrical cardioversion.^[30]^

### 4.4 Research hotpots

Keywords reflected the core theme and main content of papers, therefore they can provide a reasonable description of research hotspots.^[18]^ Based on the keywords co-occurrence map obtained before, the top 20 keywords were selected to get the top 3 research hotspots and list accordingly.

#### 4.4.1 Risk factors and stroke prevention

At present, relatively clear risk factors for AF are age, ^[31]^ race,^[32]^ and sex.^[33]^ In addition, some modifiable factors which could increase risk of AF include congestive heart failure (HF),^[34–36]^ hypertension(HTN),^[37,38]^ diabetes mellitus (DM),^[39]^ obesity,^[40,41]^ alcohol consumption,^[42–44]^ and obstructive sleep apnea (OSA).^[45]^ Behavioral interventions for AF must be based on clearly defined risk factors in order to be effective. Moreover, risk management also has important influence: stroke prevention in AF must balance the patient's risk of thromboembolism with the risk of bleeding. CHA2DS2-VASc was the most authoritative risk stratification tool recommended by the guidelines to assess the risk of stroke and bleeding.^[28,46]^

#### 4.4.2 Pathophysiology of AF

At present, scholars have not got a consensus on the specific mechanism of AF. The triggers and progression of AF appear to be a result of multiple mechanisms. In 2003, Sanders. proposed the theory of triggers of AF, a milestone in the history of AF, which included the spontaneous depolarization of atrial cells. These transient ectopic tachycardia initially reduced the atrial electrical instability, leading to atrial fibrillation.^[47]^ When AF was triggered, it was difficult to suppress if it was not treated in time (that AF begets AF).^[48]^ In the later stages of AF, atrial structural remodeling and atrial electrophysiological remodeling occurs.^[49]^ These atrial histological changes could lead to slower atrial conduction speed, local heterogeneous conduction, and local blockage. All of these are the biological substrates for AF.^[50]^ In addition, the autonomic nervous system may have a role in heterogeneous atrial electrical conduction, and may trigger atrial arrhythmias, including AF.^[51]^

#### 4.4.3 Ablation of AF

Over the past decades, catheter ablation (CA) for AF has become one of the most common heart procedures in the United States due to its safety and efficacy.^[52]^ As mentioned above, pulmonary veins, which trigger AF, are the most common anatomic source of ectopic atrial beats. The advantages of catheter PVI over antiarrhythmic drugs alone in maintaining the sinus rhythm have been demonstrated in several clinical trials.^[53–55]^ These trials reported success rates ranging from 66% to 86% at 12 months, indicating that CA was the most successful way for patients with paroxysmal AF. Favorable success rates driving the inclusion of CA in the guidelines.^[56]^ However, it should be emphasized that CA had a low ablation success rate in persistent AF and long-term AF (> at 12 months), and it was associated with frequent recurrent surgical treatment. Therefore, it was not usually for these patients.^[48]^ In addition, the evidence for whether CA can reduce the risk of stroke, heart failure, and death is still insufficient.

## 5 Conclusion

In our paper, CiteSpace and VOSviewer were used to do literature measurement on a large amount of WOSCC data. The graphics produced from this analysis show that this area of research has been growing markedly over the last 15 years and the graphs illustrate this in a relatively intuitive way. Our aim was to assess past research activities in the field of atrial fibrillation, to track their evolution and to predict new trends in specific subjects or sub-areas. Understanding these aspects is another way to help us understanding the clinical study of atrial fibrillation as well as the disease itself. The data also preliminarily revealed cooperation between authors and institutions, and may provide information for future research.

## 6 Strengths and limitations

As far as we know, this is the first bibliometric analysis of research specifically for AF. In order to fully understand the current research status of AF, 3 bibliometric tools were used to identify the countries, institutions, authors, and hotspots in this field. The data analyzed was large enough to reflect the current status of AF research. However, this study also has some limitations. First, the data were retrieved from WOSCC and only contained research papers (as opposed to reviews, meeting abstract et al). Second, since some authors used the same abbreviations of their names and some keywords could be included as multiple expressions, some accuracy may have been lost despite our efforts to standardize and correct them.

## Author contributions

**Conceptualization:** Shuqing Shi, Jingjing Shi.

**Data curation:** Shuqing Shi, Qiulei Jia.

**Formal analysis:** Shuqing Shi, Jingjing Shi.

**Investigation:** Shuqing Shi, Qiulei Jia.

**Methodology:** Shuai Shi, Guozhen Yuan.

**Project administration:** Yuanhui Hu.

**Resources:** Shuqing Shi, Jingjing Shi.

**Software:** Shuqing Shi, Dandan Wang.

**Writing – original draft:** Shuqing Shi.

**Writing – review & editing:** Shuai Shi, Yi Wei.
